# Histomorphometric analysis of the femoral neck in patients with and without femoral neck fracture

**DOI:** 10.1590/1413-78522015230201055

**Published:** 2015

**Authors:** Caio Gonçalves de Souza, Vanda Jorgetti, Luciene Machado dos Reis, Alberto Tesconi Croci

**Affiliations:** 1 Universidade de São Paulo, Faculdade de Medina, Hospital das Clínicas, Department of Orthopedics and Traumatology, São Paulo, SP, Brazil, 1. Department of Orthopedics and Traumatology, Hospital das Clínicas da Faculdade de Medina da Universidade de São Paulo, São Paulo, SP, Brazil; 2 Universidade de São Paulo, Faculdade de Medina, Laboratory of Medical Investigation, São Paulo, SP, Brazil, 2. Laboratory of Medical Investigation LIM 16, Faculdade de Medicina da Universidade de São Paulo, São Paulo, SP, Brazil

**Keywords:** Bone and bones/, Hip fractures, Bone remodeling, Osteoporosis, Densitometry

## Abstract

**OBJECTIVE:**

To determine, through bone histomorphometry in femoral neck, whether there are differences in the cancellous bone of the proximal femur from female patients over 60 years old who had femoral neck fracture and similar patients who did not have such fracture.

**METHODS:**

We analyzed the trabecular part of the femur of 13 female patients, aged over 60 years old, by the bone histomorphometry method. Seven of these patients had femoral neck fracture. All of them were subjected to hip arthroplasty.

**RESULTS:**

Bone densitometry showed no significant difference. There was no significant difference on the average thickness of the trabecular bone (124.38µm versus 147.09µm). The number of bone trabeculae was lower (1.52, versus 1.88) and the separation between them was larger (541,19µm versus 391,14µm) in the fracture group.

**CONCLUSION:**

A difference in histomorphometric parameters of cancellous bone of the femur neck was observed among patients who had fractures as compared to patients who had not.

**Level of Evidence II, Diagnostic Studies.:**

## INTRODUCTION

Nowadays injuries represent a major cause of deaths among the world population. Fractures, especially in elderly patients, lead to several clinical complications and generate a high cost to the health systems of many countries. According to statistics found in the literature, each year around three hundred thousand fractures in the proximal femur are caused by low-energy trauma, only in the United States.^1^ This type of fracture primarily affects elderly patients. The costs and difficulties in the management of these patients led us to try to prevent these fractures before they occur, and some developments have occurred in this area with medications administered to prevent bone fragility.^2^^,^ 3 However, it is still unclear on the literature what are the factors (and their chronological order) that lead to failure of the bone tissue of the proximal femur, and the exact knowledge of all of them is very important to take more effective prevention measures in the future and reduce the financial cost and even of lives that this type of fracture cause.

It is known that upon aging bones of the axial skeleton become larger in diameter in the medullary area and a less dense cortical region, which probably leads to a lower resistance to load support.^4^^,^ 5 To some authors, this fact alone is what leads to elderly patients to have a larger number of femoral neck fractures by low trauma energy.^6^^,^ 7 However, it is known that patients with this type of fractures tend to have, a few years before, fractures in the body of vertebrae. This is because the percentage of spongy bone (trabecular bone) on vertebral bodies is higher than in the femoral neck and, with aging, the cancellous bone has decreased bone density earlier that the cortical bone. Therefore, we can predict that the cancellous bone of the femoral neck must undergo structural changes before the cortical bone, and if we can see these changes (with some auxiliary noninvasive examination) before the cortical be affected, our prevention will be more effective.

The question that led to this work is why some people fracture the femoral neck and others of the same age, gender and race, do not. We believe that attention was not given to the spongy region of the proximal femur, and the reason for this is that whenever it was thought that its changes would be obvious by simply analyzing the knowledge gained from iliac crest biopsies in osteoporosis studies. If we consider the structural parameters in bone histomorphometry of the iliac crest, we know that in osteoporosis we usually find an increased trabecular separation and a reduced number of these. The thickness of bone beams is also decreased. As we know that patients with osteoporosis are at increased risk of fracturing the femoral neck, many are led to believe that in the femoral neck fracture we would find the same parameter changes. However, there is nothing definitive about this.^8^^-^ 10 Our first hypothesis, therefore, was that there is a difference in the structural parameters of the trabecular bone of the femoral neck among people who fractured and did not fracture.

In another line of thought, the spongy area of the femoral neck should undergo anatomical changes before the cortical area and, therefore, before a fracture occurs. Probably, even patients who had femoral neck fractures, but are in the age group at risk, must present these changes, even if the cortical thickness is still normal, which would allow us to make an early prevention of fractures in a phase in which the cortical is still well bearing the load and the risk is small. According to this line of thought, the results we expected to find in the histomorphometric analysis of the femoral neck would not show differences in the structural parameters (number of trabeculae, trabecular thickness and separation of these), because both groups have already suffered trabecular changes and only the changing of the cortical thickness is what differentiates the group that had mechanical failure from the one that did not.

The objective of this research was to verify, through bone histomorphometry of the femoral neck, whether there was a difference in the cancellous bone of the proximal femur in female patients over 60 years old who had femoral neck fracture and similar patients who did not have this fracture. 

## MATERIALS AND METHODS

The patients in this study were initially selected by the diagnosis at hospital admission according to the criteria of inclusion and exclusion of the research project. This research project was approved by CaPPesq and all patients signed an Informed Consent Term.

The inclusion criteria were the following:

1. Female patients, aged between 60 and 90 years old.
2. Having as admission diagnosis unilateral osteoarthrosis of the hip joint or femoral neck fracture Garden type III or IV.^11,12^
3. Indication for hip arthroplasty surgery.
4. Not presenting as medical history any endocrine treatment, use of corticosteroids, anticonvulsants or immunosuppressive drugs for over a year, or cancer diagnosis.
5. Not having, as background, orthopedic surgical treatments of the affected and contralateral hip.
6. Not performing regular physical activity, i.e. sedentary.
7. Having the cervicodiaphyseal between 130 and 140 degrees in plain radiographs of the proximal third of the contralateral femur at anteroposterior projection.

The exclusion criteria were the following:

1. Patients who refused to donate the surgical specimen (femoral head).
2. Patients who did not deambulate for more than six months.
3. Patients who used bisphosphonates for a total period (continuous or not) lasting more than six months.
4. Patients who used bisphosphonates in the last two years preceding the date of surgery.

**Chart 2.**
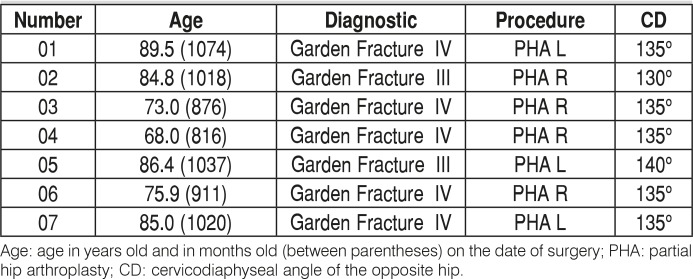
List of patients of Group 2 undergoing surgical treatment of hip, in chronological order of hospitalization, with date of admission, age (in years and in months between the parentheses), initial diagnosis, date of surgery and the procedure performed.

After analyzing the inclusion and exclusion criteria we selected 13 patients who were divided into two groups: Those in which the initial diagnosis was femoral neck fracture were included in Group 1 (fracture group). When the initial diagnosis was primary unilateral osteoarthrosis of the hip were included in Group 2 (control group). (Charts 1 and 2)

Age: age in years old and in months old (between parentheses) on the date of surgery; THA: total hip arthroplasty; CD: cervicodiaphyseal angle of the opposite hip.

**Figure:**
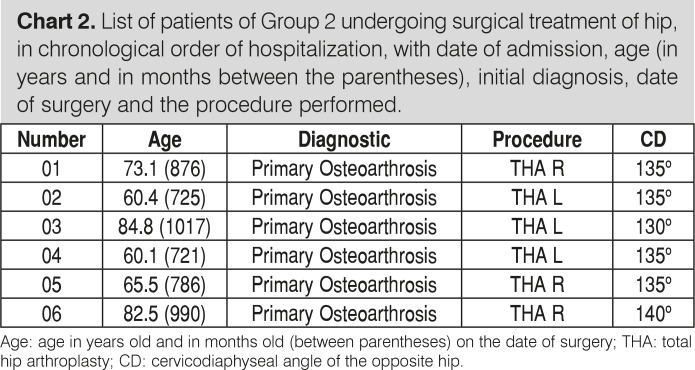

Age: age in years old and in months old (between parentheses) on the date of surgery; PHA: partial hip arthroplasty; CD: cervicodiaphyseal angle of the opposite hip.

In Group 1 we had seven patients aged between 816 months (68.0 years old) and 1074 months (89.5 years old). The mean age was 964 months and the standard deviation 95.5 months. Two patients had a Garden III fracture of the femoral neck and five had Garden IV fractures. All of them were submitted to partial hip arthroplasty. In Group 2 we had six patients aged from 721 months (60.0 years) to 1017 months (84.5 years). The mean age was 852 months and the standard deviation 130 months. All of them had a diagnosis of primary osteoarthritis of the hip and all underwent total hip arthroplasty. (Table 1)

**Table 1.** Age (in months old) on the date of surgery of patients submitted to hip arthroplasty (collection of material for bone histomorphometry).

	Group 1	Group 2
	1074	876
	1018	725
	876	1017
	816	721
	1037	786
	911	990
	1020	
Mean	964.57	852.50
Standard deviation	96.55	129.99
Standard error	36.49	53.07
Minimum	816.00	721.00
Maximum	1074.00	1017.00
Number	7	6

p value Student t-test (bicaudal): 0.115837.

In Group 1, two of them had Garden type III and five Garden type IV fractures. Regarding the affected side, four were on the right and three on the left side. The cervicodiaphyseal angle of the contralateral side varied between 130-140 degrees, with an average of 135 degrees. All underwent partial hip arthroplasty surgical treatment.

In Group 2 all were of the primary type. Regarding the affected side, three were on the right and three on the left side. The cervicodiaphyseal angle of the contralateral side varied between 130-140 degrees, with an average of 135 degrees. All underwent total hip arthroplasty surgical treatment.

Bone densitometry examination of patients was made to assess bone mineral density of the femoral neck. Patients who had done this exam up to four months before the date of the surgery did not need to repeat it. Some patients did not have the test done and did it two months after surgery. Usually the test is done in the right hip, but two patients in the fracture group made it on the left side, since the other side was operated.

**Figure 1.**
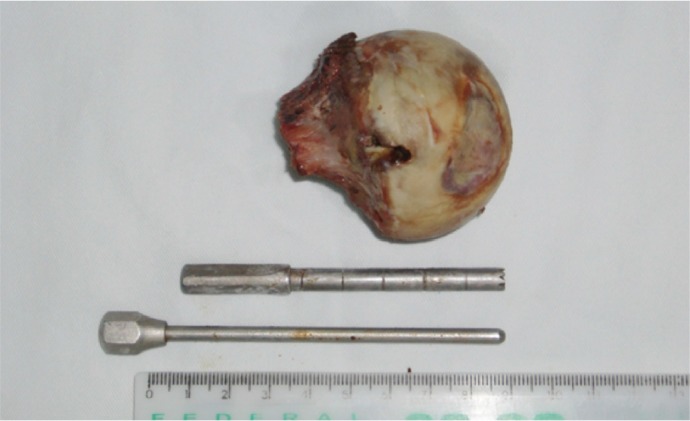
Femur head and neck next to trephine and rod.

During surgery, the femoral head was removed along with the cranial part of the neck, without surgical instrument that would damage the femoral head. (Figure 1) Surgery continued in the normal pace by the usual surgical procedure.

A trephine was made especially for this research, with a 7mm internal diameter and a plug for the electric perforator at the other end. With the head and femoral neck already out of the operative field, a bone cylinder fragment was removed exclusively by clockwise rotary movements. For the removal of this cylinder from the bone fragment it has been standardized that the trephine would always enter the location through the middle of the circumference of the femoral neck and would always be directed to the pit of the round ligament of the head. The trephine was removed by applying rotational movements in the counter-clockwise direction to avoid fragmentation of the cylinder, and later it has been removed from within the trephine by a rod of the same size of the light that has been made especially for this procedure.

In order to avoid compression of the trabeculae, the rod removed the cylinder by applying pressure on the serrated part of the cylinder (trephine) without damaging it. It is important to remember that these parameters were used in all fragments of the two groups, as this enables the analysis of cancellous bone of the femoral neck, and that the trephine did not pass by any area of bone sclerosis.

**Figure 2.**
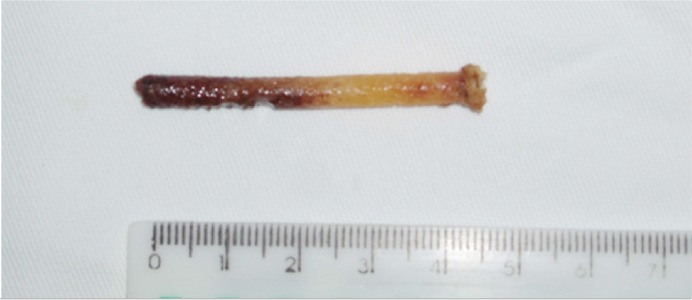
Bone cylinder before being placed in 70% ethanol.

The bone cylinder, with a length ranging from four to five centimeters, was placed in a bottle with 70% ethanol and stored for three days. (Figure 2) The cylinders were then sent to the laboratory for histomorphometric analysis.^13^

After the third day, ethanol 70% was removed and the flask was filled with 100% ethanol, where the fragment remained for three days.

The fragment was then removed from the vial and placed immersed in toluene for one day. The fragment was only three days in this solution, then 1% benzoyl peroxide was added, for three days. Then, the fragment was for further three days in a mixture of the initial solution plus 2.5% benzoyl peroxide.

After these procedures the ensemble was taken to a drying oven at 37°C until the polymerization of methyl methacrylate was reached. The blocks were cut in an impact microtome with a tungsten blade, originating histological 5 µm cuts. The sections were placed on slides and stained with 0.1% toluidine blue at pH 6.4. 

The slides were blindly analyzed by a professional and, therefore, the analysis of the regions of the blades were evaluated following purely technical criteria to ease viewing and calculation of measured parameters. The histological images were designed on a computer with the aid of a cursor on a scanner board. The method used in this study to measure the histomorphometric variables was semi-automatic, using a binocular microscope Nikon Labophot-2A(r), a video camera, a scanner board and the Osteomeasure(r) software, suitable for measurement of the parameters studied.^13^ The histomorphometric parameters measured are those standardized by the American Society of Bone and Mineral Research:

1. Trabecular Volume - BV / TV (%): volume occupied by the cancellous bone, mineralization or not, expressed as a percentage of the volume occupied by the bone marrow and trabeculae.

2. Trabecular thickness (or beams thicknesses) - Tb.Th (µM): thickness of the trabecular bone expressed in microns.

3. Trabecular separation (or beams separation) - Tb.Sp (µM): distance between the trabecular bones expressed in microns.

4. Trabecular number (or number of beams) - Tb.N (/ mm): number of bone trabeculae per millimeter of tissue, being also an index which expresses the trabecular density.

Besides these four parameters, we also calculated the total area of bone tissue that was measured and the patient's age in months, from birth to the day of surgery.

In order to compare samples of both groups we used the Student t-test. For this purpose, a value of *p*<0.05 was assumed.

The experimental hypothesis (H1) was that there was difference between the two groups. The null hypothesis was that there would be no difference. The study was considered tailed.

## RESULTS

Comparing the data on bone mineral density obtained through bone density testing, we found that the average value of the fracture group was 0.641 g/cm^2^, while the average of the control group was 0.663 g/cm^2^. (Table 2) Applying statistical analysis, we found no significant differences between them.

**Table 2.** Bone mineral density of the femoral neck measured by bone densitometry (g/cm2).

	Fracture group	Control group
	0.641	0.645
	0.604	0.636
	0.629	0.655
	0.673	0.656
	0.659	0.711
	0.655	0.677
	0.681	
Mean	0.649	0.663
Standard deviation	0.027	0.027
Standard error	0.010	0.011
Minimum	0.604	0.636
Maximum	0.681	0.711
Number	7	6

p value Student t-test (bicaudal): 0.352159.

The data found in histomorphometry showed a significant difference between groups in trabecular separation parameters and number of trabeculae. The mean trabecular separation in the fracture group was 541.19 µm in the fracture group fracture and 391.14 µm in the control group. However, the mean number of trabeculae found was lower in the fracture group, with 1.52 compared to 1.88 in the control group. On the parameters of volume occupied by the trabecular bone in relation to the bone marrow and trabecular thickness (124.38 µm* versus* 147.09 µm), no significant differences occurred. (Tables 3, 4, 5, and 6)

**Table 3.** Volume occupied by cancellous bone, mineralized or not, expressed as a percentage of the volume occupied by the bone marrow and trabeculae, measured in the region of the femoral neck by the bone histomorphometry method.

	Group 1	Group 2
	16.81	18.84
	13.01	22.06
	13.45	21.01
	28.03	32.65
	22.94	44.64
	16.60	24.15
	24.31	
Mean	19.31	27.23
Standard deviation	5.80	9.78
Standard error	2.19	3.99
Minimum	13.01	18.84
Maximum	28.03	44.64
Number	7	6

p value Student t-test (bicaudal): 0.120234.

**Table 4.** Thickness of the trabeculae expressed in µm, measured in

	Fracture group	Control group
	119.04	102.56
	100.04	139.68
	103.04	107.77
	167.17	163.39
	125.09	249.60
	115.21	119.55
	141.09	
Mean	124.38	147.09
Standard deviation	23.35	54.98
Standard error	8.83	22.45
Minimum	100.04	102.56
Maximum	167.17	249.60
Number	7	6

p value Student t-test (bicaudal): 0.377795.

**Table 5.** Thickness (separation) between bone trabeculae in µm measured in the area of the femoral neck bone by the histomorphometry method.

	Fracture group	Control group
	589.10	420.55
	668.63	493.58
	662.82	405.20
	429.20	337.00
	420.25	315.01
	578.90	375.50
	439.40	
Mean	541.19	391.14
Standard deviation	109.76	64.08
Standard error	41.48	26.16
Minimum	420.25	315.01
Maximum	668.63	493.58
Number	7	6

p value Student t-test (bicaudal): 0.012056.

**Table 6.** Number of bone trabeculae per mm of bone tissue found in the femoral neck region measured by the bone histomorphometry method.

	Fracture group	Control group
	1.40	1.99
	1.30	1.57
	1.30	1.94
	1.67	1.99
	1.83	1.78
	1.44	2.02
	1.72	
Mean	1.52	1.88
Standard deviation	0.21	0.18
Standard error	0.08	0.07
Minimum	1.30	1.57
Maximum	1.83	2.02
Number	7	6

p value Student t-test (bicaudal): 0.00685.

## DISCUSSION

Because there are no references in the literature searched on the parameters normally found in bone microarchitecture of the femoral neck, and also due to the already discussed fact that most of the authors focused on analyzing the cortical region (not the spongy) of the femoral neck in search of reasons that can lead to its rupture, there are no published data that can be compared directly to those obtained here. As previously mentioned, we had two hypotheses that could occur in this research. The first was the existence of difference in all parameters (number of trabeculae, separation between them and trabecular thickness).^8-10^ Any difference between the fracture group and the control group (for more or for less) would be accepted. The second hypothesis was the lack of difference between the groups in the analyzed parameters, which would make them similar, at least regarding the spongy part, and the only anatomical changes responsible for the mechanical failure should be that of the cortex. After analyzing the results, we found out that the difference hypothesis was confirmed by two parameters (trabecular separation and number of trabeculae) that are virtually dependent on each other, since the lower the number of bone beams in a region, the greater the distance between them, and vice versa. Therefore, obviously, if we found a difference in one of these parameters, it would also be found in the other. What surprised us was the fact there was no difference in the thickness of the trabeculae, a fact that led to statistically similar results in the volume of the spongy bone that was found.

For the groups to be considered similar, we expected that, besides the fact that patients fit the inclusion criteria, the results of hip bone densitometry were statistically similar as compared to absolute values in g/cm^2^, which in fact occurred.

Regarding age, we observe in the data presented that there was no difference between groups from birth to the date of material collection (day of surgery). All subject groups are female because the hormonal changes are earlier in this genre (menopause), which directly influences bone metabolism. If there were in male patients groups, this could affect the results, so they were not included.

The fact that some patients (in both groups) received at some point in their lives supplementation with calcium carbonate or bisphosphonate could generate criticism. To clarify any doubts that may have occurred, we should mention that none of them used alendronate for a period longer than six months, and patients who used this medication discontinued their use at least two years before the date of surgery where the material was collected. Mundy,^14 ^in a study published in 1998, states that the calcium carbonate alone does not lead to structural changes of the bone tissue, such as increased density, it only corrects bone calcium losses to other tissues from a bad nutrition.^14,15^ If we consider calcium carbonate as a bias, we should also consider nutritional factors and it would probably be unlikely to find similar groups work for any research on bone metabolism. Harrington *et al*.^16^ showed evidences that no changes occur in bone tissue leading to increased resistance by using alendronate for a period shorter than six months. McClung *et al*.^15^ showed that risedronate only began its effect on lowering hip fracture incidence after one year of continuous use. Therefore, the use of bisphosphonates and calcium for some of these patients did not affect the final outcomes of this work.

All patients (in both groups) were considered sedentary, according to data collected from medical records, therefore their bone tissue could not have been influenced by exercises to increase bone mass.^17^

Our two groups (with or without neck fracture) had similar bone mineral densities, were of the same gender, same age, and can be considered anatomically identical (same cervicodiaphyseal angle). They are, therefore, similar.

These differences, if examined by other noninvasive tests, such as quantitative tomography, may become a factor of increased risk of femoral neck fracture, making important its study.

## CONCLUSIONS

There are differences in bone microarchitecture of cancellous bone of the femoral neck in female patients older than 60 years, with or without femoral neck fracture. The differences are in the number of trabeculae, which was lower in the group of patients with fractures, and the separation between them, which is higher in patients with fracture.

There was no significant difference between the thicknesses of the trabeculae on the two groups.
